# Group B streptococcus cystitis presenting in a diabetic patient with a massive abdominopelvic abscess: a case report

**DOI:** 10.1186/1752-1947-6-237

**Published:** 2012-08-10

**Authors:** Kimberly B Ulett, Jennifer H Shuemaker, William H Benjamin, Chee K Tan, Glen C Ulett

**Affiliations:** 1 Department of Medicine, University of Alabama at Birmingham, Birmingham, AL, 35294, USA; 2 Department of Microbiology, University of Alabama at Birmingham, Birmingham, AL, 35294, USA; 3 School of Medical Sciences, and Griffith Health Institute, Centre for Medicine and Oral Health, Griffith University, Southport, 4222, Australia; 4 Present affiliation: Department of Medicine, Royal Brisbane and Women’s Hospital, Bowen Bridge Road, Herston, Queensland, 4006, Australia; 5 Department of Medicine, Royal Brisbane and Women’s Hospital, Bowen Bridge Road, Herston, Queensland, 4006, Australia

**Keywords:** Abscess, Cystitis, Group B streptococcus, *Streptococcus agalactiae*, Urinary tract infection

## Abstract

**Introduction:**

*Streptococcus agalactiae* or group B streptococcus is a Gram-positive pathogen that is typically associated with neonatal disease and infection in pregnant women. Group B streptococcus also causes invasive infections in non-pregnant adults including urinary tract infections. The spectrum of urinary tract infections caused by group B streptococcus includes cystitis, pyelonephritis, urosepsis and asymptomatic bacteriuria, which is particularly common among elderly individuals. A rare form of invasive group B streptococcus infection in adults is secondary abscess. Here, we present the first reported case of a patient who developed an unusual, massive abdominopelvic abscess secondary to acute group B streptococcus urinary tract infection.

**Case presentation:**

A 46-year-old African-American woman presented to the University Emergency Department complaining of urinary tract infection symptoms and severe abdominal pain. Diagnostic imaging by transvaginal ultrasound and computed tomography revealed a massive peripherally-enhancing, low-attenuating fluid collection within her pelvis. The patient’s abdominopelvic abscess was drained by ultrasound-guided drainage and this yielded a septic aspirate that was culture positive for abundant *S. agalactiae*. A recent history of urinary tract infection symptoms in the patient suggested that her abscess developed secondary to cystitis. Complete resolution of the abscess as a favorable outcome was achieved in this case following surgical drainage and appropriate antimicrobial therapy.

**Conclusion:**

Acute bacterial urinary tract infection leading to an abdominopelvic abscess has not previously been reported in the literature. This case report defines a new disease etiology associated with acute streptococcal cystitis and it will be of interest in cases of urinary tract infections where there is an association with abdominal and/or pelvic pain. A brief review of the literature on unusual secondary abscesses due to group B streptococcus is provided alongside this case to highlight the clinical significance and prognoses of these rare infections. Finally, this case emphasizes the requirement to distinguish unusual etiologies of pyogenic abscesses in order to guide successful clinical management and to treat patients with antibiotics active against the causal organism.

## Introduction

*Streptococcus agalactiae,* or group B streptococcus (GBS), is a leading cause of infection in newborns, pregnant women, and older persons with chronic medical illness [1]. In addition to maternal cervicovaginal colonization and neonatal infection, GBS causes invasive infections in adults including urinary tract infections (UTI). The incidence of neonatal disease has decreased due to improvements in screening and prophylaxis [1-3], and the changing spectrum of disease in adults has been noted [4]. Over 60% of cases of invasive GBS disease in the United States of America (USA) now occur in adults and most are unrelated to pregnancy [5]. A rare form of invasive GBS infection in adults is secondary abscess, which characteristically presents as a subcutaneous, retroperitoneal psoas, thoracic, aortic, or myocardial abscess secondary to an often-occult source of infection.

GBS is a notable urinary pathogen in adults [6] and reports on the incidence of genitourinary infections due to GBS have increased in the past decade [4,7-11]. The spectrum of GBS UTI includes asymptomatic bacteriuria, cystitis, pyelonephritis and urosepsis with risk factors of neurogenic bladder [12] and prior UTI [8] having been described. GBS is cultured from urine in approximately 2% of all clinically suspected cases of UTI [6,8,13]. Particularly high prevalence rates of GBS bacteriuria have been reported among elderly adults [1,14,15]. Here, we report the first case of an adult patient with an unusual, massive GBS abdominopelvic abscess that occurred secondary to cystitis, and we review prior cases of secondary GBS abscesses to describe their etiology and clinical characteristics.

## Case presentation

A 46-year-old African-American woman presented to the University Emergency Department complaining of severe abdominal pain. On presentation, she reported experiencing dysuria, urinary frequency and urgency, as well as incontinence and abdominal fullness for several weeks. Her medical history was unremarkable with no history of sexually transmitted diseases and no current medications. Her heart rate was 98, blood pressure 124/59mmHg, and her temperature was 37.6°C. Physical examination revealed increased tenderness in her right and left lower quadrants. Urine analysis revealed proteinuria, hematuria, pyuria (>50 white blood cells per high-power field), and positive urinary leukocyte esterase. A culture of the patient’s urine, which was turbid, yielded pure growth of *S. agalactiae* at 10^8^ colony forming units per liter. The patient had a hematocrit of 17%, a white blood cell count of 15.4 × 10^9^/L, glucose of 816mg/100mL, serum creatinine of 1.2mg/100mL, and HbA1C of 10.6.

**Figure 1:**
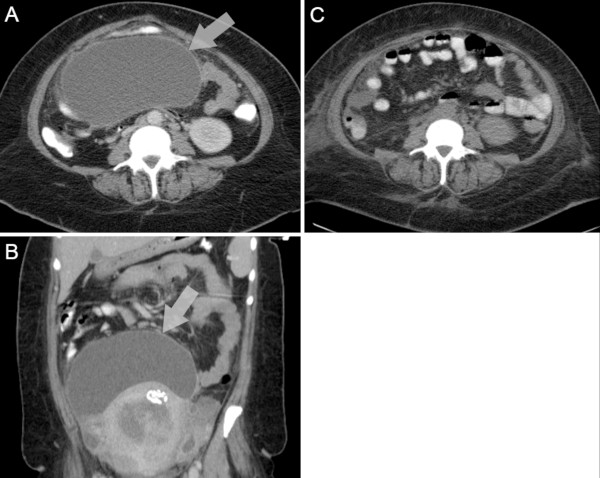
**Computed tomography scans showing a large abscess within the patient’s pelvis that was culture positive for S. agalactiae.** Transverse ( **A**) and coronal ( **B**) contrast-enhanced computed tomography scans of the patient showing a large abscess within her pelvis extending to the right adnexa (arrow) that was culture positive for *S. agalactiae.* Near complete resolution was achieved ( **C**) one day after fluoroscopic-guided abscess drainage.

A transvaginal ultrasound revealed a fibroid uterus and a heterogeneous fluid collection in the patient’s pelvis. Examination by computed tomography (CT) showed a massive peripherally enhancing, low-attenuating fluid collection measuring approximately 17.4cm by 10.4cm within her pelvis extending to the right adnexa (Figure 1). A second smaller collection was noted within the pouch of Douglas; the two fluid collections did not appear to communicate. There were several reactive inguinal, mesenteric, and retroperitoneal lymph nodes as well as subtle areas of right renal cortical non-enhancement suggestive of pyelonephritis. The bladder wall was markedly thickened with surrounding soft tissue stranding. The patient was given one dose of ceftriaxone empirically. Her antibiotic regimen was then changed to vancomycin and piperacillin/tazobactam for broad empiric coverage and she was admitted for management of her abdominopelvic abscess and UTI. She was also newly diagnosed with diabetes and placed on sliding scale insulin to manage her hyperglycemia. Her anemia was attributed to iron deficiency due to uterine leiomyomas.

A pigtail drainage catheter was placed using ultrasound and fluoroscopic guidance in order to drain the patient’s abdominopelvic abscess and this yielded a septic aspirate that was culture positive for abundant *S. agalactiae*. Antibiotic-susceptibility testing showed the isolate was resistant to tetracycline, intermediate to clindamycin and erythromycin, and sensitive to penicillin and vancomycin. A day after abscess drainage, a CT scan showed near complete interval resolution of the large pelvic fluid collection (Figure 1). The patient subsequently developed acute kidney insufficiency (creatinine 5.0mg/100mL), leucocytosis, and fever. Bacteremia was suspected, however, blood cultures taken at this time were negative. Urine analysis revealed persistent cystitis with pyuria and leukocyte esterase. Her renal failure was attributed to multifactorial acute tubular necrosis due to sepsis and vancomycin toxicity. After 4 days of vancomycin and piperacillin/tazobactam, the patient was switched to intravenous ceftriaxone (2g intravenous/24 hours) and 2 days later her fever and abdominal pain were resolved. The results of a repeat urine test were culture-negative. The patient was discharged on outpatient intravenous ceftriaxone and the pelvic drain was removed 2 weeks later. Metformin and insulin glargine treatment was commenced for diabetes management. She remained afebrile and was well on subsequent outpatient visits. Her acute kidney insufficiency resolved within 7 days of discharge (creatinine 1.5mg/100mL). An outpatient hysterectomy was planned for management of the patient’s anemia due to uterine leiomyomas.

## Discussion

GBS is a recognized cause of fatal puerperal sepsis in adults and rare invasive infections including unusual secondary abscesses and device-related infections. Sporadic cases of non-abdominopelvic secondary GBS abscesses in adults have been reported in the literature; a review using the PubMed database (from 1966 to current; English) with the terms “group B streptococcus,” and “*Streptococcus agalactiae*,” and “abscess” identified 60 cases comprising subcutaneous (23), retroperitoneal psoas (9), thoracic-aortic-myocardial (9), epidural (7), renal (3), subphrenic (2), perinephric (2), tubo-ovarian (2), suprasternal (1), adrenal (1), and prostatic (1) abscesses (number of cases in parentheses). Diabetes is recognized in many of these cases and probably represents a predisposing factor for invasive disease. Mortality is rarely reported and in almost all cases the etiology is unclear. In this case, UTI appeared to be the underlying cause of the atypical abscess in a patient who had no apparent risk factors aside from the unrecognized diabetes. There was no evidence of bladder perforation and management including abscess drainage was successful.

This case is of interest for several reasons: first, most intra-abdominal abscesses are polymicrobial in nature, and secondary abscesses are a rare form of invasive GBS infection in adults. Second, although cases of secondary bacterial abscess have been associated with bacteremia, primary suppuration, and sources of occult infection, no prior case reports have associated an abdominopelvic abscess with a preceding acute UTI. Although the cause of the abdominopelvic abscess in this case is most probably the patient’s UTI, an unidentified gynecologic infection, or potentially undetected bacteremia related to urosepsis, are also possible. There was no evidence of diverticulitis or bladder diverticula. Other etiologies of secondary abscess include appendicitis, ulcerative colitis, osteomyelitis, neoplasm, discitis, and trauma, none of which were noted in this patient. A limitation of the present study was that the GBS isolate cultured from the abdominopelvic abscess fluid was not retained after the diagnosis. Thus, a comparative molecular analysis, which is useful as a tool for identifying clonal relationships between GBS and could have defined the degree of relatedness of the two isolates cultured from the patient in this case, was not possible. In this regard, it is important to underscore the fact that we were unable to confirm with certainty the possibility that the UTI was the primary source of the abdominopelvic abscess in this patient. Several recent reports have identified aspects of the pathogenesis of acute UTI mediated by GBS [16-18], although there is no experimental evidence reported in the literature to the best of our knowledge that has associated GBS cystitis with a secondary abscess.

Finally, the antibiotic susceptibility profile of an intermediate phenotype for clindamycin and erythromycin noted in this case is atypical for GBS. GBS is sensitive to penicillin and its derivatives although it may be resistant to antibiotics used for empiric treatment of UTI such as trimethoprim-sulfamethoxazole. This illustrates the need for careful consideration of the antibiotics that are used empirically when GBS is cultured from suspected UTI.

## Conclusions

This case defines a new disease etiology associated with acute streptococcal cystitis and it will be of interest for cases of UTI associated with abdominal and/or pelvic pain. Overall, GBS is a relevant cause of secondary abscess of various etiologies in adults, particularly those with underlying medical problems. The particular etiology of pyogenic abscess guides clinical management and it is important to treat patients with antibiotics active against GBS when this organism is found to be the cause of a secondary abscess.

## Consent

Written informed consent for publication was obtained from the patient described in this case report. Ethical approval for the conduct of this work was obtained from the Institutional Review Board of University of Alabama Birmingham under approval number X070722011.

## Abbreviations

CT, Computed tomography; GBS, Group B streptococcus; UTI, Urinary tract infection.

## Misc

Kimberly B Ulett and Jennifer H Shuemaker contributed equally to this work

## Competing interests

The authors declare that they have no competing interests.

## Authors’ contribution

KBU and JHS analyzed and interpreted the patient data regarding the diagnosis, WHB and CKT performed the microbiological work, and CKT and GCU wrote the manuscript. All authors read and approved the final manuscript.
